# Supplementary material for: Forest production efficiency increases with growth temperature
Source: Nat Commun. 2020 Oct 21;11:5322. doi: 10.1038/s41467-020-19187-w (PMC7578801; doi:10.1038/s41467-020-19187-w)
Supplement: Supplementary file 1 — Supplementary Information [file 41467_2020_19187_MOESM1_ESM.pdf]

Forest production efficiency increases with growth temperature

Collalti et al.

Supplementary Materials

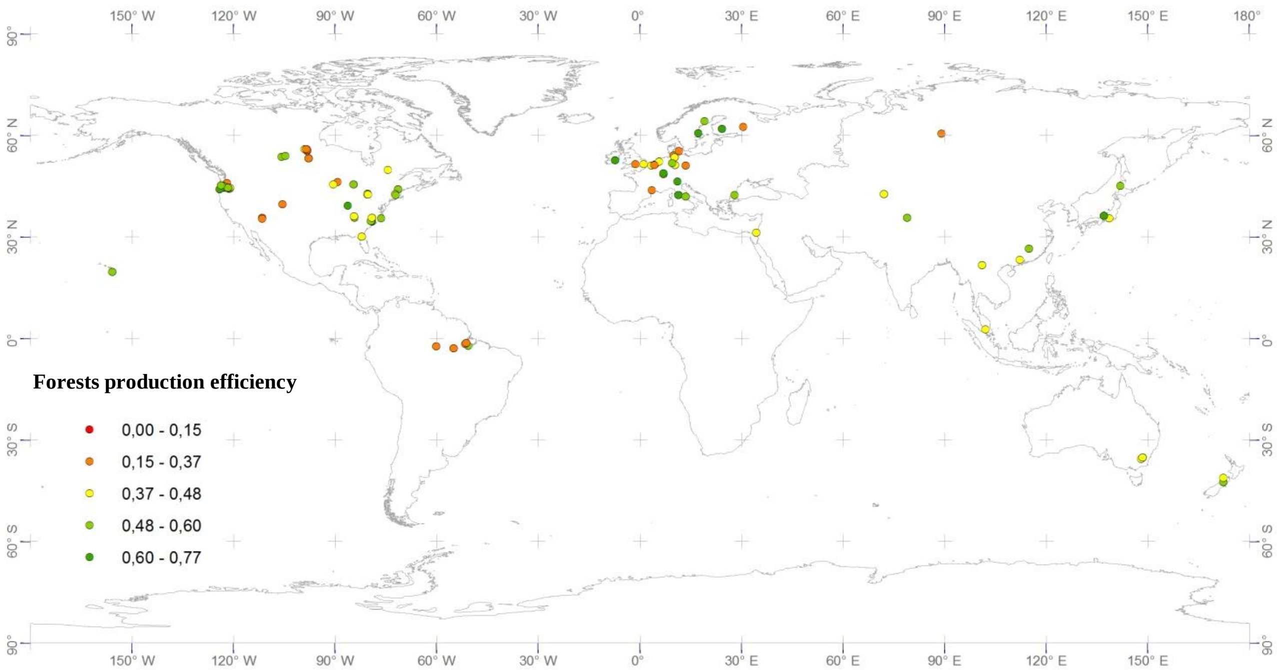

Supplementary Figure 1 | A global map of forest sites used to create a database of carbon use and biomass production efficiency (grouped as 'Forest production efficiency' in the figure legend)

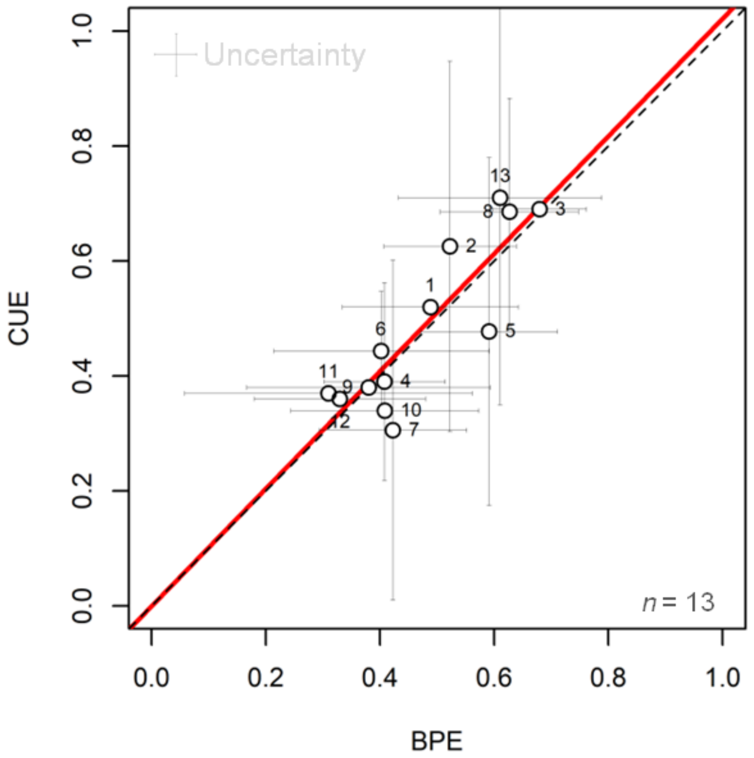

Supplementary Figure 2 | Scatter plot and linear regression line of site data where both CUE and BPE were available, forced through the origin (adjusted R^2 = 0.98, slope = 1.022 ±0.041 S.D.: i.e. not significantly different from 1, *n* = 13). The uncertainty (unitless) of the data points is indicated by bars (for data uncertainty see Methods).

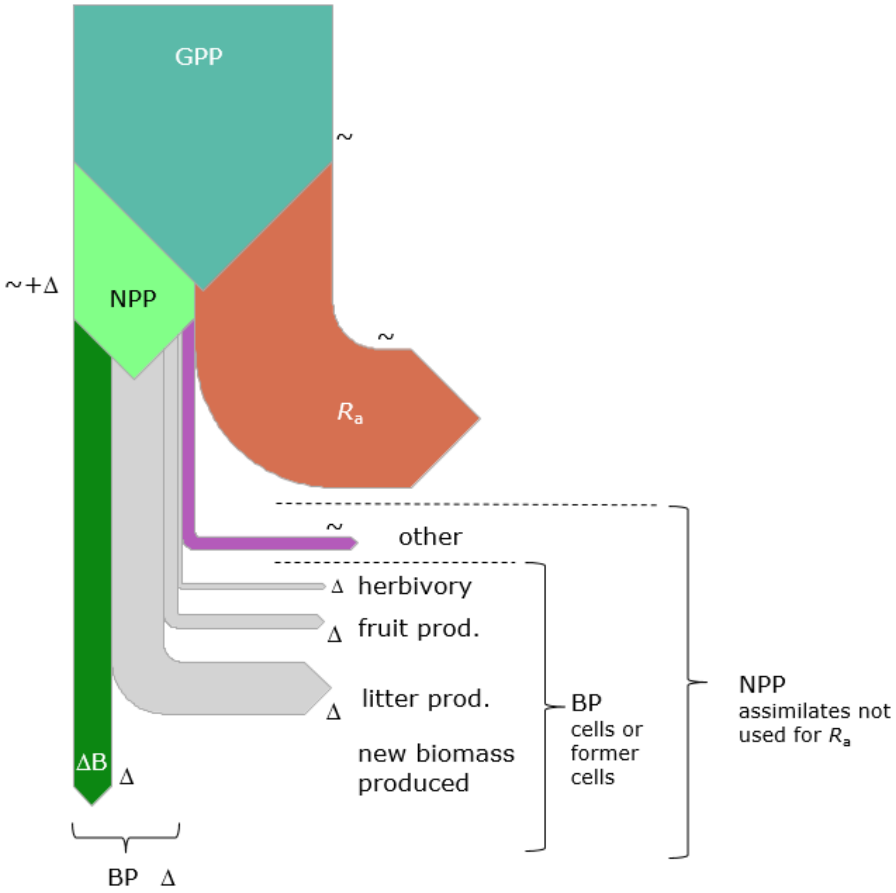

Supplementary Figure 3 | Schematic of carbon flows in and through plants, and their relationships to the quantities defined in eq. (3). Measurements are by sequential inventory (Δ) or gas and solute exchange (~) methods.

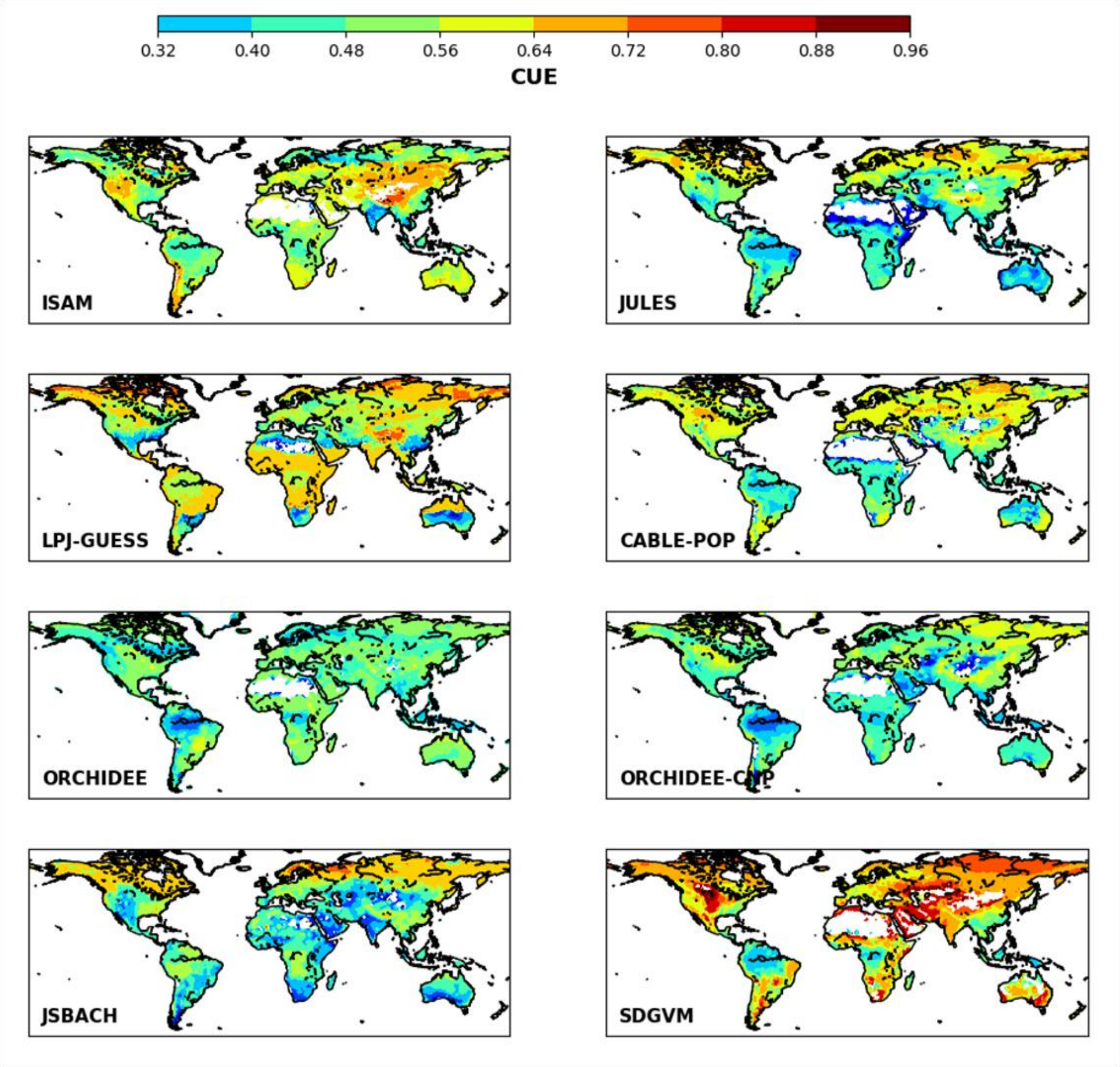

Supplementary Figure 4 | Global patterns of vegetation carbon use efficiency (CUE) derived from TRENDY v.7 process-based models: ISAM, JULES, LPJ-GUESS, CABLE-POP, ORCHIDEE, ORCHIDEE-CNP, JSBACH and SDGVM, averaged from 1995 to 2015.

Supplementary Table 1 | Pearson's correlation matrix of the model driver variables Scatter plot and linear regression line of site data where both CUE and BPE were available, forced through the origin (adjusted $R^2 = 0.98$, *slope* = 1.022 ±0.041 S.D.: i.e. not significantly different from 1).

	CUE	age	MAT	TAP	|lat|
CUE		–0.178	0.234	0.354	–0.242
age	n.s.		–0.868	–0.555	0.721
MAT	n.s.	***		0.506	–0.843
TAP	n.s.	*	*		–0.773
|lat|	n.s.	**	***	***	

Supplementary Table 2 | Fixed and random intercept variables of the models examined in step 1. The 'x' in the model matrix below represents the terms in equation (1) that include the variable of the respective column header. The intercept (β_0) is part of all models (not included in the mode matrix).

No.	MAT	age	TAP	|latitude|	random intercept GPP method
1	x	x	x	x	x
2	x	x	x		x
3	x	x		x	x
4	x		x	x	x
5		x	x	x	x
6	x	x			x
7	x		x		x
8	x			x	x
9		x	x		x
10		x		x	x
11			x	x	x
12	x				x
13		x			x
14			x		x
15				x	x
16	x	x	x	x	
17	x	x	x		
18	x	x		x	
19	x		x	x	
20		x	x	x	
21	x	x			
22	x		x		
23	x			x	
24		x	x		
25		x		x	
26			x	x	
27	x				
28		x			
29			x		
30				x	

Supplementary Table 3 | Site years with both CUE and BPE estimates

Site ID	Location	Species	Age
1	Bartlett Experimental Forest	*Acer saccarum, Fagus grandifolia, Fraxinus americana*	80
2	Bornhoved Lake Beech, Germany	*Fagus sylvatica*	111
3	Dooary forest	*Picea sitchensis*	18
4	Harvard forest	*Quercus rubra, Acr rubrum, Taxus canadensis*	100
5	Hesse, France	*Fagus sylvatica*	32
6	Hesse, France	*Fagus sylvatica*	40
7	Hyytiälä	*Pinus sylvestris*	40
8	Norunda	*Pinus sylvestris, Picea abies*	105
9	Oregon Transect cosystem Research – Metolius River Valley	*Pinus ponderosa*	148
10	Prince Albert, Canada	*Picea mariana*	115
11	Prince Albert, Canada	*Pinus banksiana*	63
12	Prince Albert, Canada	*Populus tremuloides*	68
13	Takayama, Japan	*Betula ermanii, B. platyphylla, Quercus mongolia*	40

Supplementary Table 4 | Model performance parameters for the full log-transformed model, i.e. equation (5). Parameter estimate of coefficients in equation (1) and their standard errors (Std. Error), degrees of freedoms (df), t- and p-values of the two-sided T-test and the ANOVA (* p < 0.05, ** p < 0.01, *** p < 0.001) (MAT = Mean Annual Temperature; age = stand age; TAP = Total Annual Precipitation; |lat| = absolute latitude).

	Estimate	Std Error	df	t-value	p-value	significance
Intercept						
‘Micromet’	–0.57					
‘scaling’	–0.65					
Intercept (β_0')	–0.61302	0.108603	32.5	–5.6446	2.9E–06	***
MAT (β_1')	0.006129	0.002409	132.0	2.544659	0.012089	*
Age (β_2')	–0.00042	0.000127	132.3	–3.28225	0.001317	**
TAP (β_3')	6.51E–05	2.25E–05	132.2	2.894311	0.004446	**
|lat| (β_4')	0.003737	0.001631	132.6	2.291606	0.023505	*

Supplementary Table 5 | Parameters of the mixed-effects multiple regression model (equation 1) but excluding tropical sites (i.e. |lat| < 20 degrees). Parameter estimate of coefficients in equation (1) and their standard errors (Std. Error), degrees of freedoms (df), t- and p- values of the two-sided T-test and the ANOVA (* $p < 0.05$, ** $p < 0.01$, *** $p < 0.001$) (MAT = Mean Annual Temperature; age = stand age; TAP = Total Annual Precipitation; |lat| = absolute latitude). AD- test for normality p = 0.0587.

	Estimate	Std.Error	df	p–value	Signif.
(Intercept)	0.158000	0.108	34.5	0.155245	n.s.
MAT	0.005690	2.50E-03	130	0.02428	*
age	-0.000389	1.44E-04	130	0.007723	**
TAP	0.000086	2.46E-05	130.5	0.000666	***
|lat|	0.004310	1.74E-03	130.6	0.014458	*

Supplementary Table 6 | Description of autotrophic respiration (R_a) and its components, growth (R_g) and maintenance (R_m) respiration, and reserves (non-structural carbon pool, NSC) for the eight TRENDY v.7 models used in the data vs. model comparison. For general definition of Acclimation and Adaption see Smith & Dukes (2013).

	Model							
Process	ISAM	JULES	LPJ-GUESS	CABLE-POP	ORCHIDEE	ORCHIDEE-CNP	JSBACH	SDGVM
GPP	Farquhar	Collatz	Haxeltine & Prentice	Farquhar, extended to account for co-ordination of rate-determining steps in photosynthesis.	Farquhar	Farquhar	Farquhar	Farquhar
Growth Respiration* (growth respiration coefficient, r_G)	Not available	25%	33%	>15%: magnitude depends on leaf P/N ratio	28%	28%	25%	25%
Maintenance Respiration (Temperature dependence)	Fixed Q_{10}	Fixed Q_{10}. Bell-shaped function with peak rates at 32°C	Constant modified Arrhenius function in response to temperature	Variable Q_{10} as in ref. [2]	Constant modified Arrhenius function in response to temperature	Constant modified Arrhenius function in response to temperature	Constant exponential response to temperature plus high-temperature inhibition	Stem and root: exponential increase, leaf (day): exponential increase capped at 30 °C, leaf (night): none
Maintenance Respiration (Biomass dependence)	Not available	Depends on biomass N concentration	Depends on biomass N concentration	Depends on biomass N concentration	Depends on biomass N concentration	Depends on biomass N concentration	Linear dependence on leaf area index, but LAI not depending on biomass	Stem and root: proportion of live biomass**, leaf (day): proportion of leaf N*, leaf (night): proportion of leaf N
Acclimation of R_a	Not available	Previous 10 days temperature (only for leaves)	No	Previous three months temperature (for leaves, stems and fine roots)	No	No	No	No
Adaptation of R_a	Not available	No	No	No	No	No	No	No
Autotrophic Respiration	Sum of $R_g + R_m$	Sum of $R_g + R_m$	Sum of $R_g + R_m$	Sum of $R_g + R_m$	Sum of $R_g + R_m$	Sum of $R_g + R_m$	Sum of $R_g + R_m$	Sum of $R_g + R_m$
NPP	NPP = (GPP–R_m) (1–r_G)	NPP = (GPP–R_m) (1–r_G)	NPP = (GPP–R_m) (1–r_G)	NPP = (GPP–R_m) (1–r_G)	NPP = (GPP–R_m) (1–r_G)	NPP = (GPP–R_m) (1–r_G)	NPP = (GPP–R_m) (1–r_G)	NPP = (GPP–R_m) (1–r_G)
Reserves	Not available	No	No	No	Yes	Yes	Yes	Yes
References	[4], [5]	[1], [3]	[11]	[8]	[6]	[7]	[9], [10]	[12]

* R_g = (GPP – R_m) * r_G

** but also multiplied by soil water limitation scalar

PRISMA 2009 Flow Diagram

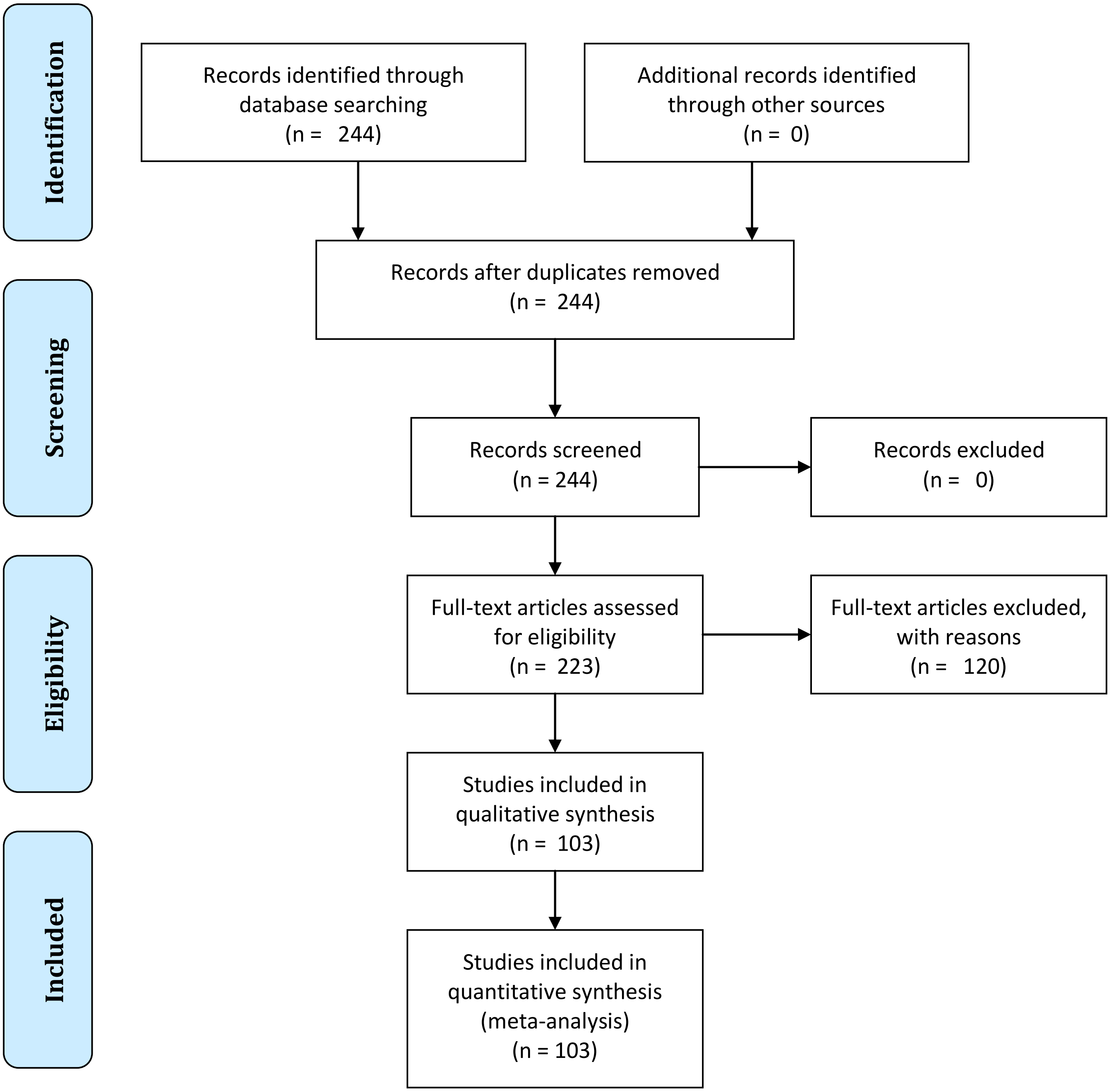

References

[1] Atkin O. K. *et al*. (2008). Using temperature-dependent changes in leaf scaling relationships to quantitatively account for thermal acclimation of respiration in a coupled global climate–vegetation model. *Global Change Biology*, 14, 2709–2726, doi: 10.1111/j.1365-2486.2008.01664.x

[2] Atkin, O. K. *et al*. (2016). Global variability in leaf respiration in relation to climate, plant functional types and leaf traits. *New Phytologist*, 211, 1142–1142.

[3] Huntingford C. *et al*. (2017). Implications of improved representations of plant respiration in a changing climate. *Nature Communications*, doi: 10.1038/s41467-017-01774-z

[4] Kheshgi, H. S., and A. K. Jain, (2003) Projecting future climate change: Implications of carbon cycle model inter-comparisons, *Global Biogeochem. Cycles*, 17(2), 1047, doi:10.1029/2001GB001842

[5] King A. W. *et al*. (1995). In search of the missing carbon sink: a model of terrestrial biospheric response to land-use change and atmospheric CO_2, *Tellus B: Chemical and Physical Meteorology*, 47:4, 501-519, DOI:10.3402/tellusb.v47i4.16064

[6] Krinner G. *et al*. (2005). A dynamic global vegetation model for studies of the coupled atmosphere-biosphere system. *Global Biogeochemical Cycles*, doi:10.1029/2003GB002199

[7] Goll D.S. *et al*. (2017). A representation of the phosphorus cycle for ORCHIDEE (revision 4520). *Geosci. Model Dev*.,

[8] Haverd V. *et al*. (2018). A new version of the CABLE land surface model (Subversion revision r4601) incorporating land use and land cover change, woody vegetation demography, and a novel optimisation-based approach to plant coordination of photosynthesis. *Geosci. Model Dev*., 11, 2995–3026, https://doi.org/10.5194/gmd-11-2995-201

[9] Mauritsen T. *et al*. (2019). Developments in the MPI-M Earth System Model version 1.2 (MPI-ESM1.2) and Its Response to Increasing CO_2, *Journal of Advances in Modeling Earth System*, 11, https://doi.org/10.1029/2018MS001400

[10] Raddatz T. J., *et al*. (2007), Will the tropical land biosphere dominate the climate-carbon cycle feedback during the twenty-first century?, *Clim. Dyn*.,29, 565–574

[11] Smith B. *et al*. (2014). Implications of incorporating N cycling and N limitations on primary production in an individual-based dynamic vegetation model. *Biogeosciences*, doi:10.5194/bg-11-2027-2014

[12] Woodward F.I. *et al*. (1995). A global land primary productivity and phyto-geography model, *Global Biogeochemical Cycles*, https://doi.org/10.1029/95GB02432
