# Supplementary material for: Forest production efficiency increases with growth temperature
Source: Nat Commun. 2020 Oct 21;11:5322. doi: 10.1038/s41467-020-19187-w (PMC7578801; doi:10.1038/s41467-020-19187-w)
Supplement: Supplementary file 3 — Reporting Summary [file 41467_2020_19187_MOESM3_ESM.pdf]

nature research

Corresponding author(s): Andreas Ibrom

Last updated by author(s): Apr 20, 2020

Reporting Summary

Nature Research wishes to improve the reproducibility of the work that we publish. This form provides structure for consistency and transparency in reporting. For further information on Nature Research policies, seeAuthors & Referees and theEditorial Policy Checklist .

Statistics

For all statistical analyses, confirm that the following items are present in the figure legend, table legend, main text, or Methods section.

n/a	Confirmed	
☐	☒	The exact sample size (*n*) for each experimental group/condition, given as a discrete number and unit of measurement
☐	☒	A statement on whether measurements were taken from distinct samples or whether the same sample was measured repeatedly
☐	☒	The statistical test(s) used AND whether they are one- or two-sided<br>*Only common tests should be described solely by name; describe more complex techniques in the Methods section.*
☐	☒	A description of all covariates tested
☐	☒	A description of any assumptions or corrections, such as tests of normality and adjustment for multiple comparisons
☐	☒	A full description of the statistical parameters including central tendency (e.g. means) or other basic estimates (e.g. regression coefficient) AND variation (e.g. standard deviation) or associated estimates of uncertainty (e.g. confidence intervals)
☐	☒	For null hypothesis testing, the test statistic (e.g. *F*, *t*, *r*) with confidence intervals, effect sizes, degrees of freedom and *P* value noted<br>*Give P values as exact values whenever suitable.*
☒	☐	For Bayesian analysis, information on the choice of priors and Markov chain Monte Carlo settings
☐	☒	For hierarchical and complex designs, identification of the appropriate level for tests and full reporting of outcomes
☒	☐	Estimates of effect sizes (e.g. Cohen's *d*, Pearson's *r*), indicating how they were calculated

Our web collection on statistics for biologists contains articles on many of the points above.

Software and code

Policy information about availability of computer code

Data collection	Publicly available data bases and publications augmented with g3graph analyzer version 1.5.2 (https://github.com/pn2200/g3data)
Data analysis	R version 3.6.2 (2019-12-12) with package versions: "lme4", 1.1.21, "lmerTest", 3.1.2 and "nortest", 1.0.4.

For manuscripts utilizing custom algorithms or software that are central to the research but not yet described in published literature, software must be made available to editors/reviewers. We strongly encourage code deposition in a community repository (e.g. GitHub). See the Nature Research guidelines for submitting code & software for further information.

Data

Policy information about availability of data

All manuscripts must include a data availability statement. This statement should provide the following information, where applicable:

- Accession codes, unique identifiers, or web links for publicly available datasets
- A list of figures that have associated raw data
- A description of any restrictions on data availability

Provide your data availability statement here.

Field-specific reporting

Please select the one below that is the best fit for your research. If you are not sure, read the appropriate sections before making your selection.

☐ Life sciences ☐ Behavioural & social sciences ☒ Ecological, evolutionary & environmental sciences

For a reference copy of the document with all sections, see nature.com/documents/nr-reporting-summary-flat.pdf

Ecological, evolutionary & environmental sciences study design

All studies must disclose on these points even when the disclosure is negative.

Study description	We collected a large list of observations of annual forest Gross Primary Productivity (GPP) , Net Primary Productivity (NPP) or total biomass production (BP) estimates, together with a set of covariates. The number of observations are explicitly mentioned for each type of analysis. The main analysis is based on mixed linear modeling with a subset of 142 data, i. e. all records with complete information on GPP, NPP or BP, mean annual air temperature , stand age, latitude and the method, with which GPP was estimated. We also used , for comparison simulation data from the TRENDY project, which was provided from the TRENDY simulations data base manager.
Research sample	we used all available publications that provided full annual estimates of GPP
Sampling strategy	Literature study
Data collection	Data are taken either from published data tables, supplementary data bases and some are taken from published figures using the G3data software. This was done by first author Alessio Collalti.
Timing and spatial scale	We used annual climate and BP and NPP and GPP data from forests (published at ecosystem scale ~ 1 km^2) from all biomes with forests (biome and global scale).
Data exclusions	1. The database includes all data that was found, except from those references that cited data from original literature, when the data from the original reference was already included. This was done to avoid statistical bias.<br>2. For the FPE/BPE/CUE density plots we used all data from the database.<br>3. For the comparison of CUE and BPE we used only those records from the data base that provided both CUE and BPE for a forest site.<br>4. For mixed linear modeling, we used all those records from the database that included empirically determined values for the variables to calculate FPE and the four driver variables MAT, TAP, age and absLat, except one record that calculated GPP from up-scaled autotrophic respiration and biometrically measured NPP (Curtis, P. S. et al. Respiratory carbon losses and the carbon-use efficiency of a northern hardwood forest, 1999–2003. New Phytol. 167, 437–456 (2005).), because of to few cases (1) to build an additional class.
Reproducibility	not relevant as we did not conduct experiments
Randomization	not relevant as we used all available data contrary to, e.g., clinical trials, where one designs a sample.
Blinding	blinding was not relevant for our study. Data ad model selection followed clearly defined protocols, which are described in the manuscript.

Did the study involve field work? ☐ Yes ☒ No

Reporting for specific materials, systems and methods

We require information from authors about some types of materials, experimental systems and methods used in many studies. Here, indicate whether each material, system or method listed is relevant to your study. If you are not sure if a list item applies to your research, read the appropriate section before selecting a response.

Materials & experimental systems

n/a	Involved in the study
☒	☐ Antibodies
☒	☐ Eukaryotic cell lines
☒	☐ Palaeontology
☒	☐ Animals and other organisms
☒	☐ Human research participants
☒	☐ Clinical data

Methods

n/a	Involved in the study
☒	☐ ChIP-seq
☒	☐ Flow cytometry
☒	☐ MRI-based neuroimaging
